# Genome size evolution in the Archaea

**DOI:** 10.1042/ETLS20180021

**Published:** 2018-11-14

**Authors:** Siri Kellner, Anja Spang, Pierre Offre, Gergely J. Szöllo˝si, Celine Petitjean, Tom A. Williams

**Affiliations:** 1 School of Earth Sciences, University of Bristol, Bristol BS8 1TQ, U.K.; 2 NIOZ, Royal Netherlands Institute for Sea Research, Department of Marine Microbiology and Biogeochemistry, and Utrecht University, P.O. Box 59, NL-1790 AB Den Burg, The Netherlands; 3 Department of Cell and Molecular Biology, Science for Life Laboratory, Uppsala University, SE-75123, Uppsala, Sweden; 4 MTA-ELTE Lendület Evolutionary Genomics Research Group, 1117 Budapest, Hungary; 5 Department of Biological Physics, Eötvös Loránd University, 1117 Budapest, Hungary; 6 School of Biological Sciences, University of Bristol, Bristol BS8 1TQ, U.K.

**Keywords:** evolution, genomics, archaea

## Abstract

What determines variation in genome size, gene content and genetic diversity at the broadest scales across the tree of life? Much of the existing work contrasts eukaryotes with prokaryotes, the latter represented mainly by Bacteria. But any general theory of genome evolution must also account for the Archaea, a diverse and ecologically important group of prokaryotes that represent one of the primary domains of cellular life. Here, we survey the extant diversity of Bacteria and Archaea, and ask whether the general principles of genome evolution deduced from the study of Bacteria and eukaryotes also apply to the archaeal domain. Although Bacteria and Archaea share a common prokaryotic genome architecture, the extant diversity of Bacteria appears to be much higher than that of Archaea. Compared with Archaea, Bacteria also show much greater genome-level specialisation to specific ecological niches, including parasitism and endosymbiosis. The reasons for these differences in long-term diversification rates are unclear, but might be related to fundamental differences in informational processing machineries and cell biological features that may favour archaeal diversification in harsher or more energy-limited environments. Finally, phylogenomic analyses suggest that the first Archaea were anaerobic autotrophs that evolved on the early Earth.

## Introduction

One of the major challenges in evolutionary genetics is to explain the enormous variation in genome size and gene content across the tree of life in terms of basic evolutionary processes such as mutation, genetic drift and selection. Phylogenomics suggests that the deepest split in the universal tree lies between the two prokaryotic domains, Bacteria and Archaea, with eukaryotes evolving more recently through a symbiosis between the two [1–4]. But despite the evolutionary and ecological importance of the Archaea, they have not been widely considered [5] in evolutionary genetic accounts for the origins of biodiversity. Until recently, few archaeal genomes were available, and it has been difficult to determine whether Bacteria and Archaea share a common prokaryotic evolutionary regime, or if instead there are conserved differences in the evolutionary processes and macroevolutionary trajectories of the two prokaryotic groups. A better understanding of archaeal evolutionary genetics will be essential for making sense of evolution at the broadest scales, and for testing hypotheses about the differing evolutionary trajectories of prokaryotic and eukaryotic cells [6,7].

In recent years, tremendous progress in the use of cultivation-independent sequencing techniques has greatly improved genomic sampling of both Bacteria and Archaea, and has led to the discovery of major new lineages in the tree of life [8–10]. This wealth of new genomic data allows us to revisit previous work on archaeal comparative genomics and to better characterise the features and processes of archaeal genome evolution. We compare the diversity of modern archaeal and bacterial genomes in terms of genome size and organisation, and ask whether the general principles of genome evolution deduced from the study of other lifeforms also apply to the archaeal domain. In Bacteria and eukaryotes, lifestyle has a profound impact on genome evolution, with the genomes of symbionts and parasites often experiencing extensive remodelling and reduction. We evaluate whether archaeal symbionts and parasites, including recently described ultrasmall lineages [9,11,12], evolve in the same way. Finally, we review comparative genomic insights into long-term trends in archaeal genome evolution and the nature of the earliest Archaea.

### A common prokaryotic genome architecture in Bacteria and Archaea

**Figure 1.**
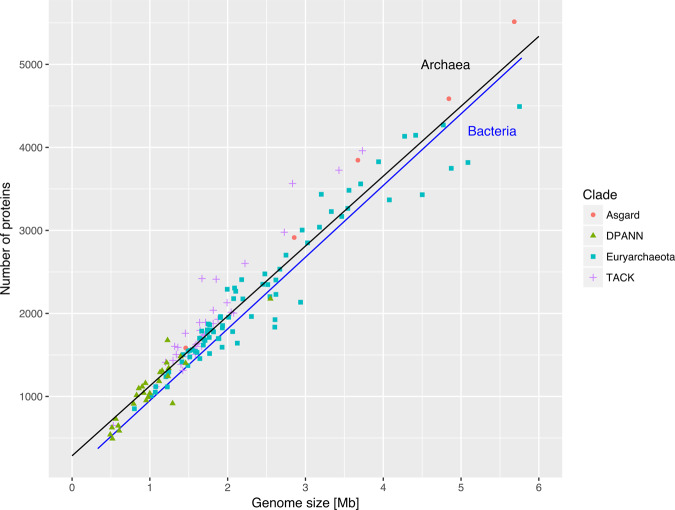
A common gene-dense genome architecture in Bacteria and Archaea. Major archaeal clades are distinguished by shape and colour. The near-linear relationship between genome size and the number of encoded proteins may reflect efficient selection against the accumulation of nonfunctional DNA in prokaryotes [13].

**Figure 2.**
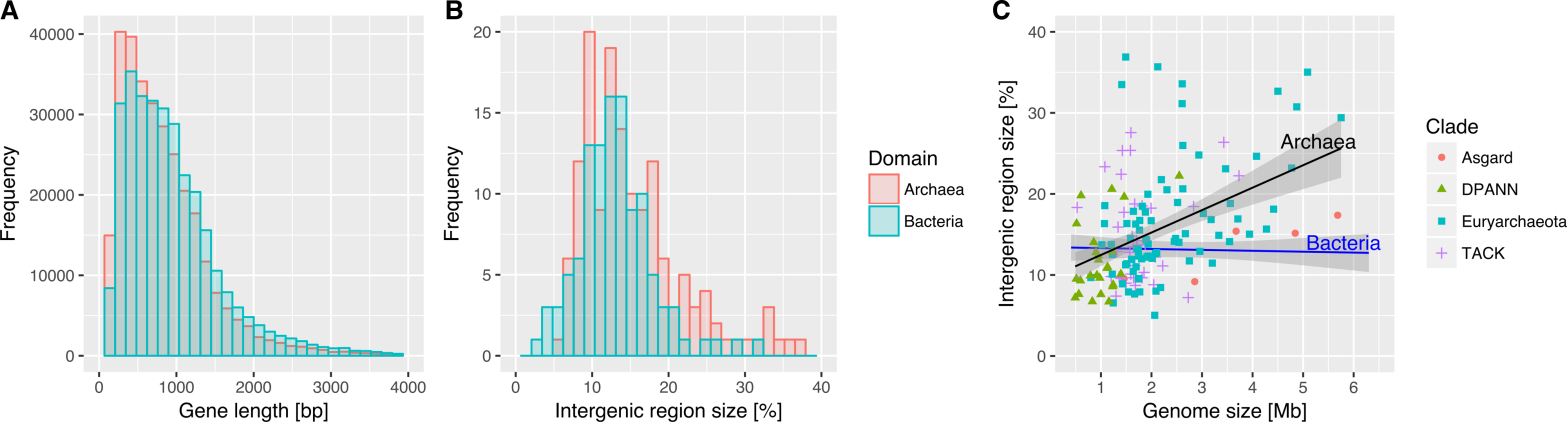
Genome composition in Bacteria and Archaea. Distributions for the lengths of (**a**) genes and (**b**) intergenic regions are broadly similar among prokaryotes, with somewhat longer intergenic regions in sampled Archaea. (**c**) The relationship between genome size and proportion of intergenic material. In Archaea, as in eukaryotes, larger genomes contain a higher proportion of intergenic material (*P* = 2.79 × 10^−5^, phylogenetic least-squares regression), although this does not appear to be the case for Bacteria.

Ten years ago, Koonin and Wolf [5] compared the genome structure and evolution of Bacteria and Archaea using the genomes then available. They concluded that archaeal and bacterial genomes share a common structure, with a main circular chromosome, high gene density, absence of introns and relatively short intergenic spaces [5]. The expanded sample of prokaryotic genomes now available confirms several of Koonin and Wolf's results, including the high gene density and low non-coding content of both archaeal and bacterial genomes (Figures 1 and 2). This common gene-dense prokaryotic genome architecture is thought to arise convergently from the generally high effective population sizes of prokaryotes, which permit efficient selection against the accumulation of nonfunctional or parasitic DNA [13], as well as a bias towards deletions during DNA replication [14,15]. The relatively low absolute numbers of genes encoded by bacterial and archaeal genomes, in contrast, has been proposed to reflect the diminishing adaptive returns of new genes as the number of existing genes in the genome increases [16]. It is interesting to note that genome architectural similarities between Bacteria and Archaea are likely to ultimately reflect similar selective regimes rather than similar molecular biology, because the DNA replication machineries of the two domains are largely non-homologous [17].

Despite the close overall similarity in bacterial and archaeal genome architectures, some differences are also apparent. In Bacteria, the proportion of the genome consisting of intergenic regions is relatively constant across a broad range of genome sizes, while in Archaea — as in eukaryotes [13] — larger genomes contain a greater proportion of intergenic material (Figure 2c). In Archaea [18] and eukaryotes [6], but not Bacteria [16], genome size appears to correlate negatively with the strength of selection. This relaxation of selection is often invoked to explain the proliferation of ‘selfish' DNA, such as introns and transposable elements, in the large genomes of multicellular eukaryotes [19]. It is tempting to speculate that a similar process might be at work among the larger archaeal genomes; unfortunately, we still know relatively little about the diversity of selfish DNA in Archaea, and, while characterised families (such as insertion sequences [20]) do vary in abundance among closely related Archaea [21], they do not appear to be more abundant in the larger genomes [18].

**Figure 3.**
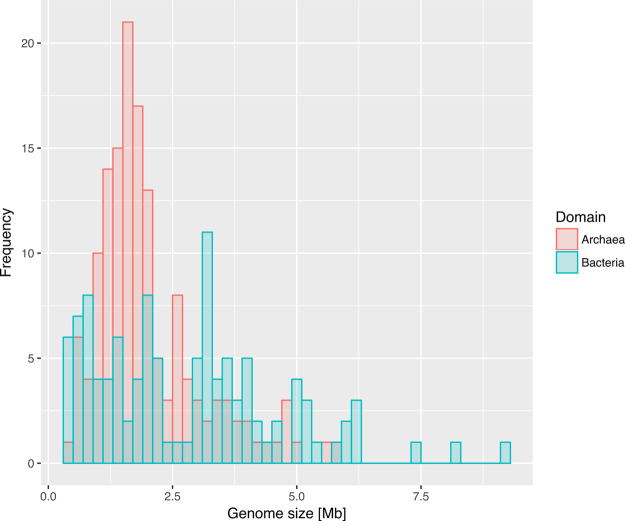
The distribution of genome sizes among sequenced Archaea (red) and Bacteria (blue). Archaea has a unimodal peak at 1.6 Mb, whereas Bacteria show a bimodal peak at ∼1.2 and ∼3.2 Mb. The size distribution of sampled bacterial genomes has a long tail, extending to at least 14.7 Mb[25], although these outliers do not change the overall distribution. Distributions calculated from a representative sample of Bacteria and Archaea drawn evenly from across the known diversity from recent phylogenomic surveys [8,26].

A second key difference between bacterial and archaeal genomes [5] is the range of genome sizes observed for the two groups. Koonin and Wolf reported that bacterial genome sizes were distributed bimodally, while archaeal genomes were distributed around a single, lower mean. Although Koonin and Wolf argued that this pattern might be explained by a bias towards sequencing parasitic and symbiotic Bacteria, it appears to hold across the much larger range of both bacterial and archaeal diversity now available (Figure 3). The unimodal distribution of genome sizes in Archaea suggests that characterised archaeal symbionts and parasites have not experienced the same degree of reductive genome evolution as in Bacteria; the underlying evolutionary basis for these differences remains unclear. Furthermore, the variation of genome size in Archaea appears to be an order of magnitude less than in bacteria[5]; characterised bacterial genome sizes vary ∼100-fold, from 0.112 Mb (*Nasuia deltocephalinicola*) to 16.04 Mb (*Minicystis rosea*), while archaeal genomes vary ∼10-fold, from 0.49 Mb (the ectosymbiont *Nanoarchaeum equitans*) up to 5.75 Mb (*Methanosarcina acetivorans*).

The smallest genomes from Archaea and Bacteria belong to parasites or symbionts, but free-living members of both groups from nutrient-limited habitats such as open marine waters also possess small genomes. Some of the most abundant ocean bacteria (*Prochlorococcus*, SAR11) are characterised by genomes below 2 Mb [22,23], and the same trend is seen in Archaea: while marine Thaumarchaeota can have genome sizes as low as 1.23 Mb (*Nitrosopelagicus brevis*), characterised relatives from terrestrial environments have genomes ranging up to 3.43 Mb (*Nitrocosmicus oleophilus*). Thus, in marine ecosystems, low nutrient availability may select for minimal genomes and metabolisms [22].

The above patterns are based on a single representative genome for each archaeal ‘species', but we know that bacterial genome sizes and gene contents can vary substantially over short evolutionary distances; for example, sequenced *E. coli* isolates vary in size by ∼20%, from 4.56 to 5.7 Mb [24]. There are few Archaea for which multiple closely related genomes are available; the best-studied case is the crenarchaeon *Sulfolobus islandicus*, which also shows some degree of size variation among the eight completely sequenced isolates (2.47–2.85 Mb)*.* More genomes from closely related Archaea will be needed to evaluate how within-species variation compares between the two prokaryotic groups.

### Genome evolution of host-associated Archaea

Symbioses — mutualistic, commensal and parasitic relationships between organisms [27,28] — are abundant in nature and can have profound consequences for genome evolution. In Bacteria and eukaryotes, the trend is typically toward significant reductive evolution of symbiont genomes — including the loss of genes and pathways needed for a free-living lifestyle [29]. In some cases, this can lead to the complete disintegration and subsequent replacement of the symbiont [30]. However, this extensive reductive evolution is predominantly seen in obligate, vertically transmitted intracellular symbionts: genome sizes of symbionts vary greatly and may even increase when compared with close free-living relatives [31]. This testifies to many different evolutionary trajectories for the size and content of symbiont genomes depending on factors such as the life history of the symbiont and its transmission mode, and may help to make sense of the patterns observed for the genomes of archaeal symbionts.

**Figure 4.**
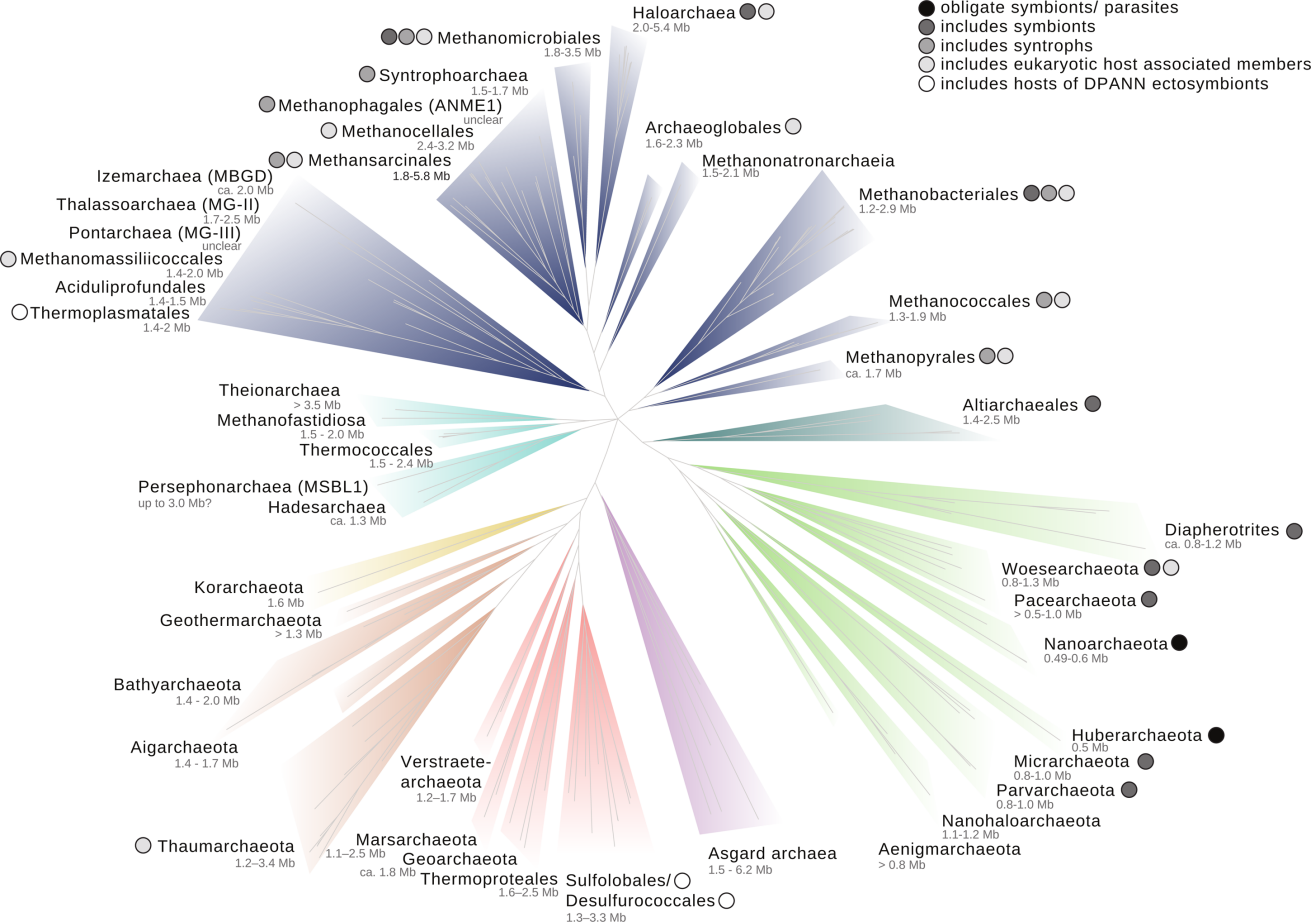
Distribution of host-associated lineages across the tree of Archaea. Host-associated lifestyles have evolved repeatedly within the Archaea. Members of unlabelled groups are assumed to be free-living, as it is currently unknown whether they engage in symbiotic associations. Black numbers denote clades where the genome size ranges are based on complete genomes; grey numbers denote approximate ranges derived from metagenome bins and/or single-celled genomes. The backbone tree is derived from a maximum likelihood analysis (82 concatenated single-copy orthologues, LG + G+F in IQ-Tree [33], some uncertain relationships near the root (for *Theionarchaea*, *Methanofastidiosa*, *Persephonarchaea* and *Thermococcales*) have been collapsed.

Very little is known about genome evolution in archaeal symbionts, which is in part due to our limited knowledge of archaeal symbiotic diversity. Many potentially symbiotic, host-associated Archaea have been reported, most notably among members of the Euryarchaeota, Thaumarchaeota and DPANN superphylum [32], suggesting that host-associated lifestyles have evolved repeatedly within the Archaea (Figure 4).

Symbiotic and host-associated Euryarchaeota include methanogens and anaerobic hydrocarbon-oxidising Archaea (ANME and Syntrophoarchaea), as well as halophiles. For example, various methanogens engage in syntrophic interactions with different bacteria and anaerobic fungi [34–36] or form part of the gut microbiome of a large range of animals including humans [37]. Some methanogens and haloarchaea are also ecto- and endosymbionts of diverse anaerobic protists [37–39] where mutualistic interactions were suggested. Several clades within the ammonia-oxidising Thaumarchaeota have consistently been detected in marine sponge and coral microbiomes [40–42] and comprise several putative symbionts — some of which may be transmitted vertically via the larvae [43]. These symbioses appear to be mutualistic or commensal, in which the archaeal symbionts contribute to the detoxification of nitrogen waste products of the host [44,45].

Perhaps the currently most striking and best-understood example of an archaeal host-symbiont system comprises the ectoparasite *N. equitans* and its crenarchaeal host *Ignicoccus hospitalis* [46]. *N. equitans* is dependent on various metabolites from *Ignicoccus* and lowers host proliferation, but does not apparently cause sustained damage [47]. Single cell and metagenomics approaches have recently led to the discovery of various additional clades of ultrasmall genome-reduced Archaea [11,12]. Thus far, phylogenies have suggested that these Archaea may form a monophyletic ‘DPANN' superphylum which also includes Nanoarchaeota [11,12,48], although phylogenetic artefacts may erroneously group some archaeal lineages within the DPANN [49]. Limited metabolic gene repertoires suggest that DPANN Archaea may be dependent on symbiotic interactions with other organisms [11]. In particular, electron microscopy and co-occurrence analyses have revealed that DPANN members Parva- and Micrarchaeota are commonly found in association with Thermoplasmatales-related hosts, while Huberarchaea may be ectoparasites of Altiarchaea [50–54], which themselves include symbionts [55]. Altogether, this indicates that DPANN comprises a largely unexplored diversity of potential novel archaeal ecto- or perhaps even endosymbionts.

### Genomic features of archaeal symbionts?

While systematic and comparative studies of genomic features of archaeal symbionts are lacking, currently available genomes of methanogens, ANME and Syntrophoarchaea cover a relatively broad range of sizes (0.49–5.8 Mb) indicating that the ability of some Archaea to take part in syntrophic interactions is not characteristically associated with a reduced genome and proteome. Much remains to be learned about the molecular and cellular traits involved in the various known syntrophic interactions [35], and although the general features defining archaeal syntrophs remain poorly characterised, recent work has indicated that large multi-heme *c*-type cytochromes mediate syntrophic interactions in Archaea [56].

The most genome-reduced Archaea belong to the DPANN superphylum among which the ectoparasitic Nanoarchaeota and Huberarchaea have the smallest known genomes. For example, the genomes of *N. equitans* [57] and its close relative *Nanopusillus acidilobi* [58] are only 490 and 606 kb in size, respectively, and lack genes for various anabolic and catabolic pathways including a functional ATP synthase [58,59]. In general, members of the DPANN have genomes ranging from ∼0.5 to 1.5 Mb in size and many representatives lack genes for central carbon and energy metabolism. In contrast with bacterial endosymbionts, however, the DPANN genomes have a surprisingly high coding density and very few pseudogenes [9] and — despite their sparse metabolic gene repertoires — have retained genes for informational processing machinery ([57], confirmed by a new analysis in Supplementary Table S1). Both DPANN and Asgard archaea encode many uncharacterised proteins, but this enrichment of information processing over metabolism is restricted to DPANN, and might therefore be a hallmark of parasitic or symbiotic lifestyles (Supplementary Table S1). Interestingly, diversity generating retroelements, which contribute to rapid and targeted mutations in specific target genes through reverse transcription, are overrepresented in DPANN genomes [60–62]. While the effects of these elements are target-specific, their activity may underlie the accelerated evolutionary rates seen for at least some proteins in this group. Despite these initial insights, much has to be learned about DPANN genome evolution and the link between reduced genome sizes and putatively symbiotic lifestyles.

### Are there obligate archaeal endosymbionts?

While an archaeal partner played an important role in the evolution of the eukaryotic cell by the acquisition of a bacterial endosymbiont [1,63,64], other examples of Archaea engaging in obligate relationships with a bacterial or eukaryotic partner are unknown thus far. Although some methanogens form intracellular symbioses with anaerobic protistan hosts, it is not known whether these Archaea are obligate symbionts. Erosion of some of the proteinogenic amino acid biosynthetic pathways in these methanogens might indicate a step toward host dependence [65], but extensive genome reduction has not been observed so far. This is in contrast with the multitude of obligate intracellular bacteria reported from eukaryotic hosts, some of which display extreme genome reduction [66].

### Why are archaea less diverse than bacteria?

The consensus is that the root of the tree of life lies between the Bacteria and Archaea [67–71], but comprehensive phylogenetic surveys suggest that the diversity of modern Bacteria is much greater than that of the Archaea — as well as of eukaryotes [8,9]. One possibility is that current sampling or sequencing methods provide a biased view of bacterial and archaeal diversity [72]. While this is certainly the case for 16S rRNA-based surveys, it is less of a concern for single cell and in particular for metagenomic approaches, the latter of which target environmental DNA directly [73] and thus circumvent the biases inherent to primer based approaches. Setting aside the potential technical issues, we do not currently have a good explanation for why bacterial and archaeal diversity should be so different, and — since Bacteria and Archaea collectively make up most of life's genetic diversity — this represents a major gap in our understanding of how biodiversity evolves.

One possibility is that the universal root is not between the Bacteria and Archaea. If the root was within the Bacteria, this would provide more time for the accumulation of among-lineage bacterial diversity. Several root positions within Bacteria have been suggested [74–76], but none have received wider support. What little evidence is available from the fossil and geochemical record suggests that Archaea are likely to be quite old, perhaps originating before 3.5 Gya [77,78]. While interpretation of such ancient biomarkers is fraught with difficulty, the antiquity of the Archaea is also supported by recent molecular dating studies combining evidence from gene transfers and relaxed molecular clocks [79,80], with the last archaeal common ancestor (LACA) likely having evolved prior to 3.51 Gya [79,80].

If Bacteria and Archaea are both ancient lineages, then differences in their extant genetic diversity must reflect differences in long-term evolutionary rate, in terms of either mutation rates and selective pressures, or different macroevolutionary histories. Little is known about mutation rates in Archaea, and to the best of our knowledge mutation accumulation data are available for just a single archaeon, the thermophile *Sulfolobus acidocaldarius* [81]. Similar to thermophilic bacteria, the estimated per-base mutation rate in *Sulfolobus* is among the lowest reported for cellular life [82]. Drake [83] proposed that low mutation rates in thermophiles might be selectively advantageous because the average effect of a new mutation is expected to be more deleterious in harsh environments. Conceptually, this shift in the distribution of fitness effects might be thought of as a transition to a rugged fitness landscape in which it is unusually difficult to cross the valleys between the adaptive peaks representing ecotypes or species. Thus, at least for thermophiles, a shift in the distribution of fitness effects might explain both selection for a lower mutation rate and lower long-term rates of diversification [84]. Although most Archaea are not thermophiles, it is tempting to apply this line of reasoning more broadly, because adaptation to harsh conditions of other kinds — particularly energy stress, low energy flux and extremes of pH — have been suggested to be a common feature shared across the archaeal domain [85]. These hypotheses will remain speculative until more data on mutation rates and the distribution of fitness effects in Archaea inhabiting a broad variety of habitats become available.

### Archaeal genome evolution in deep time

The antiquity of the Archaea has led to substantial interest in early archaeal evolution and the nature of LACA, with the aim of providing insight into the metabolisms of the earliest lifeforms and the environments that supported life on the early Earth. As might be expected given the enormous timescales involved, inferences of LACA's genome size and gene content are uncertain, and published estimates vary depending on the reconstruction methods used. Csuros and Miklos [86] used a phylogenetic birth–death model to study the evolution of gene family profiles via gene gain, duplication and loss along a candidate species tree. This method did not use information from the gene phylogenies, but was instead based upon counts of homologous genes on each genome. Analyses under this model suggested that gene loss outnumbered gene gain on most branches of the archaeal tree, so that genomic streamlining from a relatively complex common ancestor was suggested to represent the dominant mode of archaeal genome evolution [86,87].

**Figure 5.**
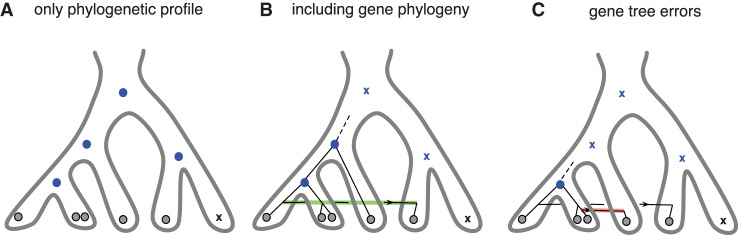
Ancestral reconstruction using only phylogenetic profiles can lead to artefactually large ancestral gene contents. Grey circles denote observed genes in the genomes of present-day organisms; blue circles and crosses denote inferred ancestral presence or absence in ancestral genomes. (**a**) The phylogenetic profile of this gene family is consistent with presence at all ancestral nodes, with a single loss in the branch leading to one of the modern lineages. (**b**) The gene family tree indicates that the gene was not present in the common ancestor; instead, it originated later in evolution, but was subsequently transferred into the right-hand side of the species tree. (**c**) An important caveat of introducing phylogenetic information in the form of gene family trees is that, while it mitigates the systematic bias of profile only methods in overestimating the number of genes in ancestral genomes, it can also lead to an underestimation of the number of genes in ancestral gene contents if errors are present in the gene phylogeny. Here, an incorrect inference of gene transfer places the origin of the gene too recently in the species tree. As discussed in the main text errors in the gene phylogeny can be greatly reduced using species tree aware methods.

One potential limitation of profile-based approaches is that, without information from the individual gene trees, there is very limited power to detect horizontal gene transfer unless the gene family has an extremely patchy phylogenetic distribution [88]. As illustrated in Figure 5a,b, this can lead to a systematic overestimation of the number of genes in ancestral genomes [48,86]. This limitation has motivated the development of models that extend the birth–death approach by explicitly considering information from the gene trees, leading to probabilistic gene tree-species tree reconciliation [89]. The main advantage of using species-tree aware gene tree reconstruction methods such as ALE, is that conditional on the species tree being correct, these methods produce dramatically more accurate gene trees [89–92], and correspondingly fewer gene transfer events. This reduction in the number of spurious transfer events caused by errors in the gene tree ameliorates the problem of underestimating the number of genes in ancestral genomes (Figure 5c).

Williams et al. [48] used one such method, ALE [89], to model gene family evolution on the archaeal species tree. In contrast with profile only analyses, the results supported a scenario in which archaeal gene content has gradually increased through time, with *de novo* gene origination, duplication and transfer generally outweighing gene loss. In this analysis, LACA was inferred to have encoded 1090 gene families, rising to ∼1500 families among modern Archaea. In this regard, it is important to note that gene tree reconciliation methods (Figure 5) outperform profile only methods, because they are able to distinguish the ancestral gain and subsequent recurrent loss of a gene family from more recent gain followed by gene transfer. As a result, they tend to infer larger rates of transfer and more realistic ancestral genome sizes [48,88]. Thus, the inference of a large ancestral genome in LACA followed by reductive evolution can be explained by the very poor power to detect gene transfer in the absence of evidence from gene tree topologies, and the corresponding systematic inflation of ancestral genome sizes. While some doubt over the ancestral genome size remains, these and other analyses suggest that the Wood–Ljungdahl pathway may have been the earliest carbon fixation pathway in the Archaea [48,93,94], supporting the view that LACA was an anaerobic autotroph.

## Conclusions

Archaeal genome sequencing has lagged behind that for Bacteria, and until the advent of environmental genomics — and the resulting data deluge from abundant but uncultivated microorganisms — it was unclear whether the observed differences between archaeal and bacterial genomes reflected sampling artefacts or biological differences between the domains. Here, we took advantage of the much broader sample of available prokaryotic genome diversity, which allowed us to include genomes representing a broad range of habitats and lifestyles. Our analyses confirm early indications [5] suggesting that, while genome architecture is conserved between Bacteria and Archaea, there appear to be important differences characterising the genomic diversity of the two domains, whether measured in terms of sequence divergence or variation in genome size and coding capacity. The greater genomic malleability of Bacteria is particularly evident for symbionts and parasites: symbiotic Archaea are varied and ecologically important, but — with the important exception of the archaeal host for eukaryote origins — they do not generally experience genome reduction to the same extent as their bacterial counterparts.

The general patterns now seem clear, but we still lack a mechanistic understanding of the evolutionary forces that underlie them. Developing that understanding will require more data from at least two sources. First, we still know very little about the biology and environmental interactions of the many new lineages of Archaea (and indeed Bacteria) that have recently been sequenced using environmental genomics. A more detailed understanding of their lifestyles, and of variation in lifestyle within groups such as the DPANN Archaea, will be critically important in interpreting the broad-scale patterns we have reviewed here. Secondly, testing hypotheses about mutation, selection and diversification will require estimates of the mutation rate and distribution of fitness effects from representative lineages sampled across the archaeal tree, but particularly from mesophilic Archaea. The increasing interest from a broad range of researchers in archaeal genomics and biology, and new techniques for genome-informed cultivation and the study of microbial metabolisms, may now provide the opportunity to begin to explore these questions.

## Summary

- Archaea and Bacteria share common gene-dense prokaryotic genome architecture.
- The range of archaeal genome sizes is much narrower than that of Bacteria. There are many ecologically important archaeal parasites and symbionts, but they are not as extremely reduced as their bacterial counterparts.
- Archaea appear to be as old as Bacteria, but their extant diversity is much lower. We do not know why this is the case.
- The first Archaea were likely anaerobic autotrophs that lived on the early Earth. Their genomes were probably modestly smaller than those of extant Archaea.

## Abbreviations

LACA: last archaeal common ancestor
